# Supplementary material for: Structure and function of the SIT1 proline transporter in complex with the COVID-19 receptor ACE2
Source: Nat Commun. 2024 Jun 29;15:5503. doi: 10.1038/s41467-024-48921-x (PMC11217458; doi:10.1038/s41467-024-48921-x)
Supplement: Supplementary file 1 — Supplementary Information [file 41467_2024_48921_MOESM1_ESM.pdf]

Supplementary information for

Structure and function of the SIT1 proline transporter in complex with the COVID-19 receptor ACE2

Huanyu Z. Li[1], Ashley C.W. Pike[1], Irina Lotsaris[2], Gamma Chi[1], Jesper S. Hansen[1], Sarah G. Lee[3], Karin E.J. Rödström[1], Simon R. Bushell[1], David Speedman[1], Adam Evans[1], Dong Wang[1], Didi He[1], Leela Shrestha[1], Chady Nasrallah[1], Nicola A. Burgess-Brown[1], Robert J. Vandenberg[2]*, Timothy R. Dafforn[3]*, Elisabeth P. Carpenter[1]*, David B. Sauer[1]*

[1] Centre for Medicines Discovery, Nuffield Department of Medicine, University of Oxford, Oxford, UK

[2] Molecular Biomedicine Theme, School of Medical Sciences, University of Sydney, Sydney, Australia

[3] School of Biosciences, University of Birmingham, Birmingham, U.K.

*Correspondence to: R.J.V. (robert.vandenberg@sydney.edu.au), T.R.D. (t.r.dafforn@bham.ac.uk), E.P.C. (lizcarpen1@gmail.com), D.B.S. (david.sauer@cmd.ox.ac.uk)

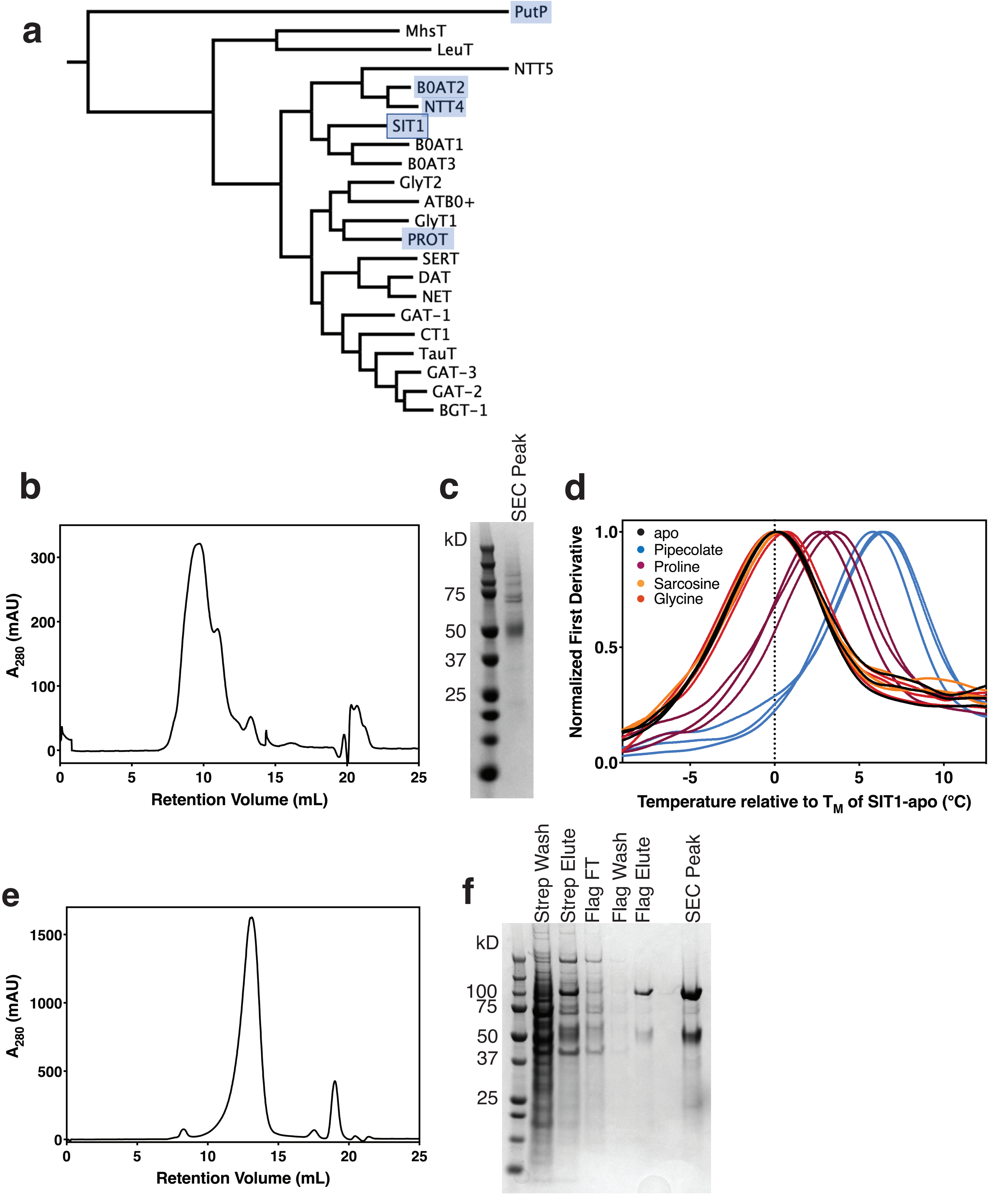

Supplementary Figure 1. Purification of SIT1 and the ACE2-SIT1 complex. (**a**) Phylogenetic tree of human SLC6 proteins and bacterial homologs, with proline transporters highlighted. (**b**) Preparative size-exclusion chromatography of SIT1 on a Superdex 200 Increase 10/300 GL column. (**c**) Representative SDS-PAGE of SIT1 purification from 10 biological replicates. (**d**) Thermostabilization of detergent solubilized SIT1 by various amino acids. (**e**) Preparative size-exclusion chromatography of the ACE2-SIT1 complex on a Superose 6 Increase 10/300 GL column. (**f**) Representative SDS-PAGE for the purification of the ACE2-SIT1 complex from 10 biological replicates.

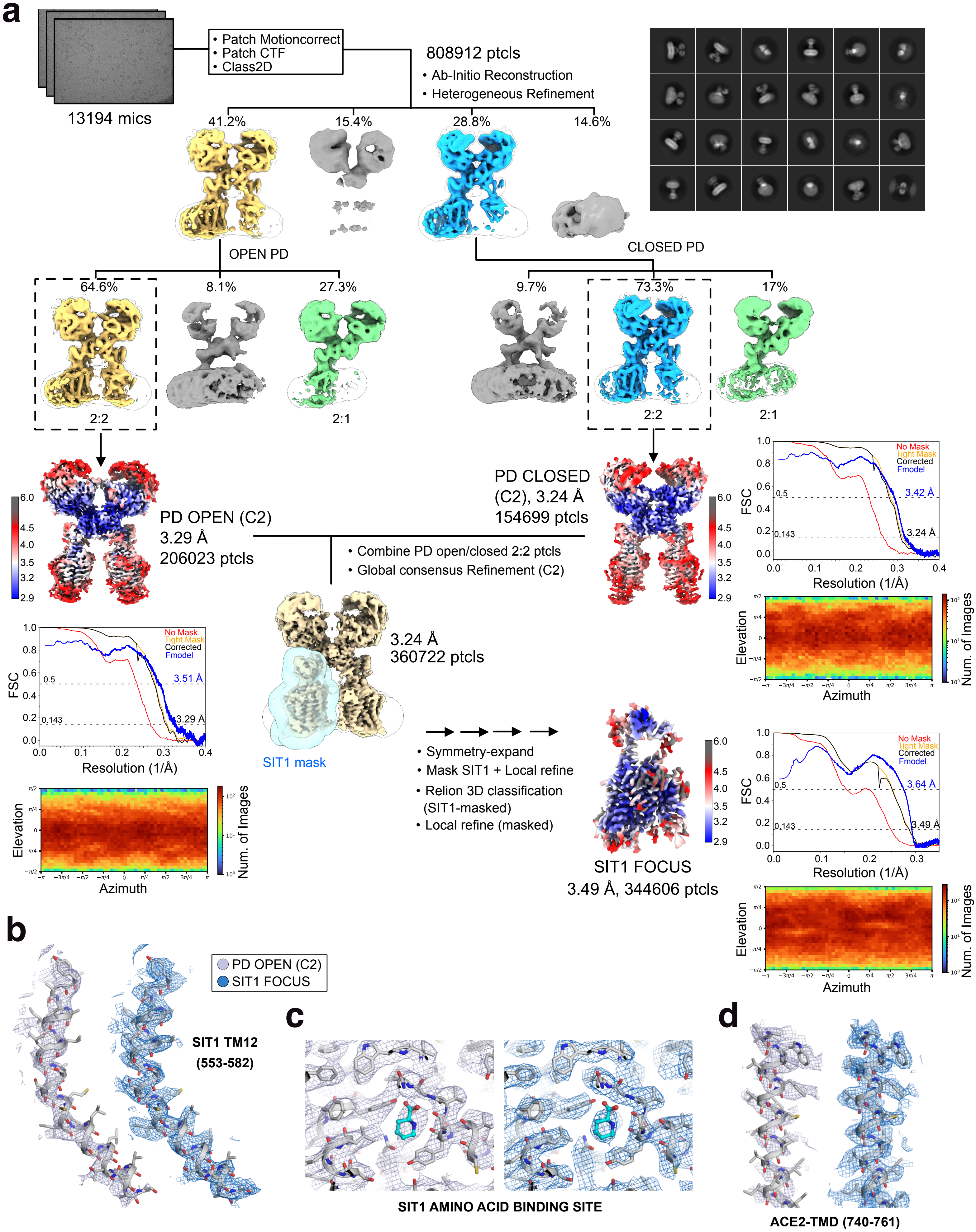

Supplementary Figure 2. ACE2-SIT1 structure determination. (**a**) Flow chart for determining the structure of the ACE2-SIT1 complex in the presence of pipecolate. Representative densities for (**b**) SIT1's TM12, (**c**) SIT1's amino acid binding site, and (**d**) ACE2's TMD. Coulombic potential maps are shown in grey and blue respectively for the PD open map with C2 symmetry imposed and the SIT1 focused refinement, respectively.

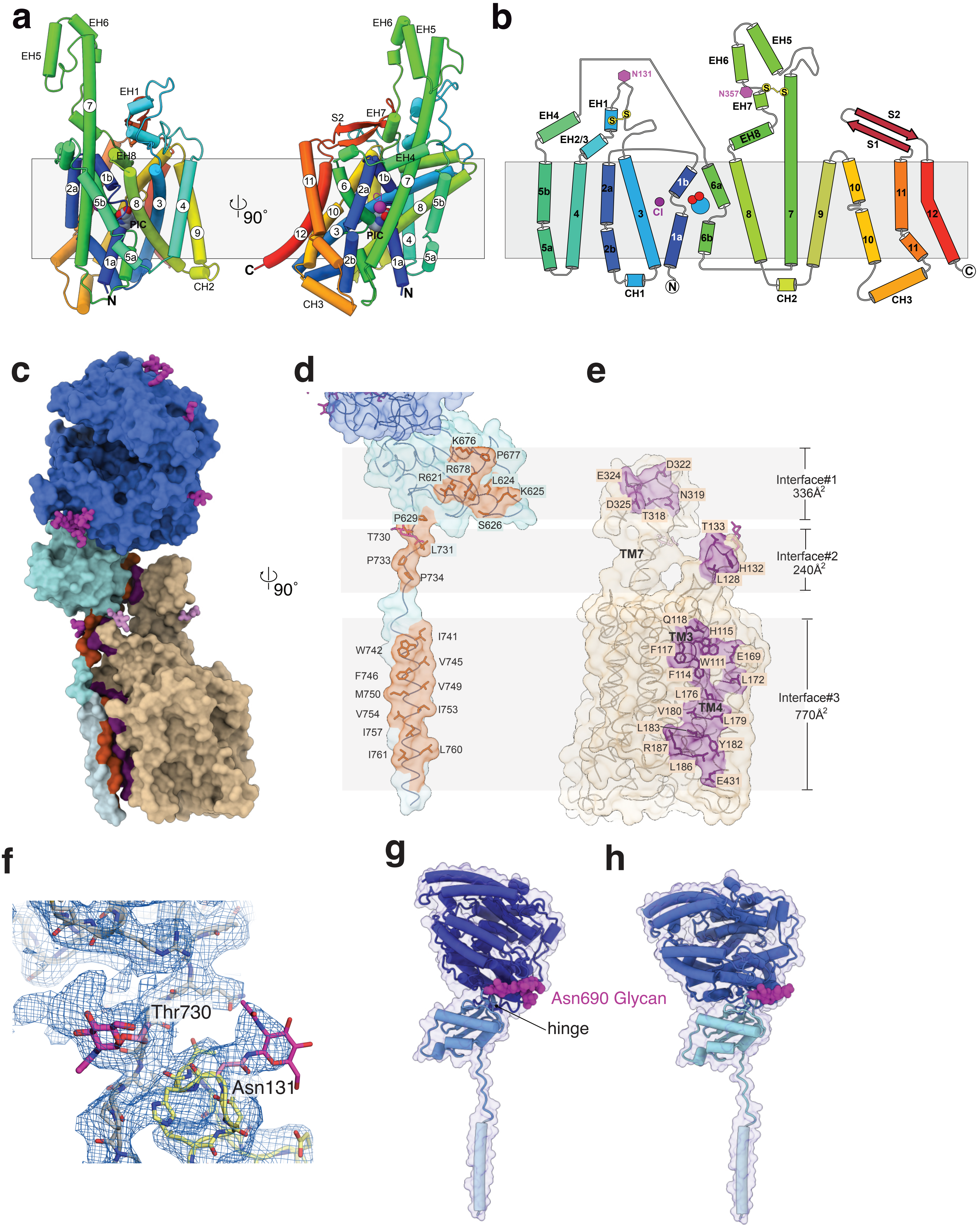

Supplementary Figure 3. ACE2-SIT1 interactions. SIT1's (**a**) heterodimeric structure and (**b**) topology. (**c**) ACE2-SIT1 dimeric structure. ACE2 and SIT1 colored in blues and wheat, respectively. Open book representation for dimer interface residues of (**d**) ACE2 and (**e**) SIT1. ACE2 and SIT1 interface residues colored in orange and purple, respectively. (**f**) O-linked glycosylation at Thr730. ACE2 structure, SIT1 structure, and Coulombic potential map colored in grey, yellow, and blue, respectively. ACE2 structure viewed from the plane of the membrane (**g**) open and (**h**) closed conformation. Glycan at Asn690 shown as magenta spheres.

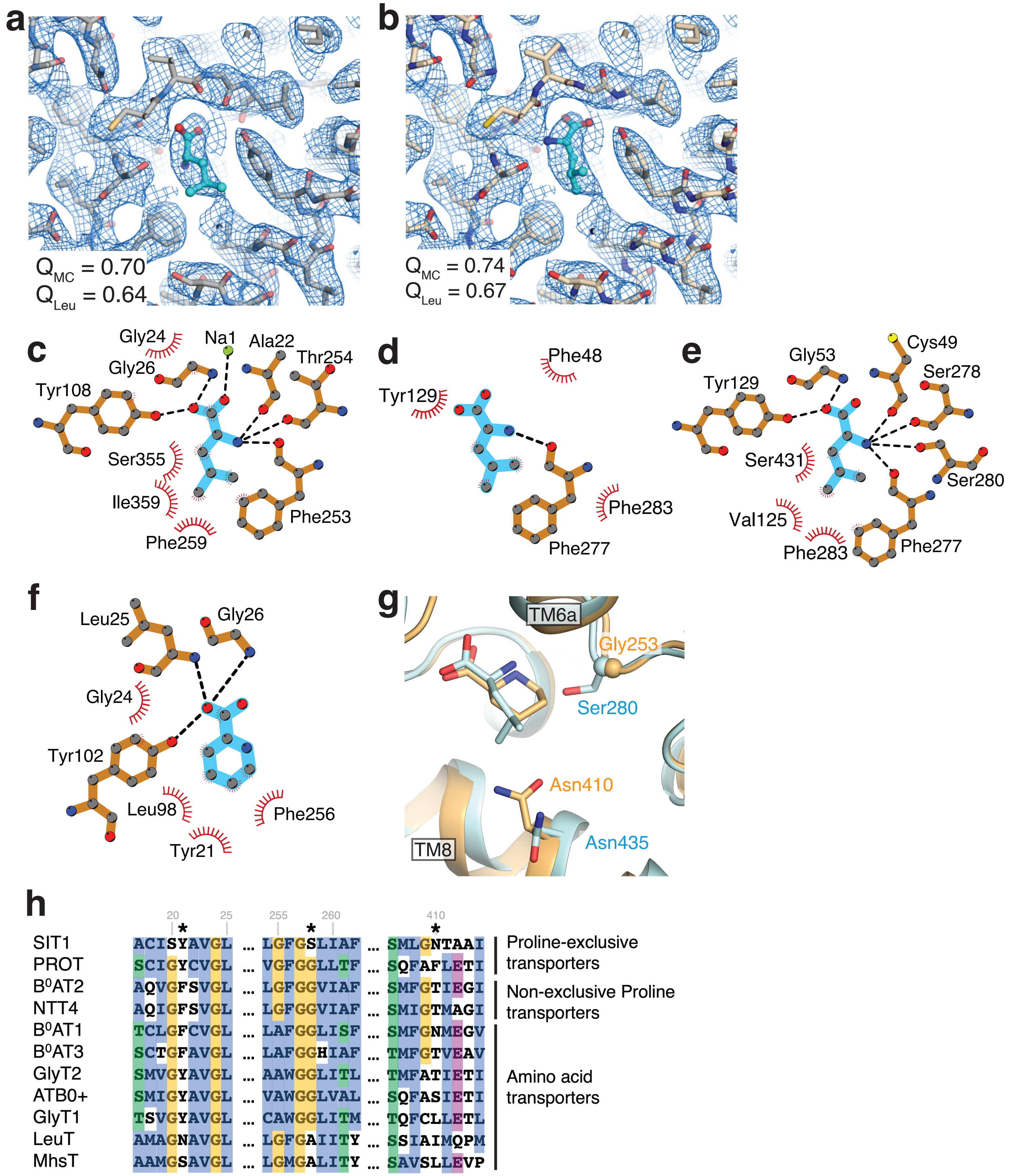

Supplementary Figure 4. SLC6 amino acid interactions. (**a**) Published structure of leucine bound B^{0}AT1 (6M17) with map-to-model fit scores for the main chain and leucine substrate. (**b**) Refit structure of leucine bound B^{0}AT1 with map-to-model fit scores for the main chain and leucine substrate. Contact maps for (**c**) leucine-bound LeuT, (**d**) published leucine-bound B^{0}AT1 (6M17), (**e**) refit leucine-bound B^{0}AT1, and (**f**) pipecolate bound SIT1. (**g**) Overlay of pipecolate bound SIT1 and leucine-bound B^{0}AT1, colored in wheat and blue, respectively. (**h**) Sequence alignment for SLC6 amino acid transporters and homologs.

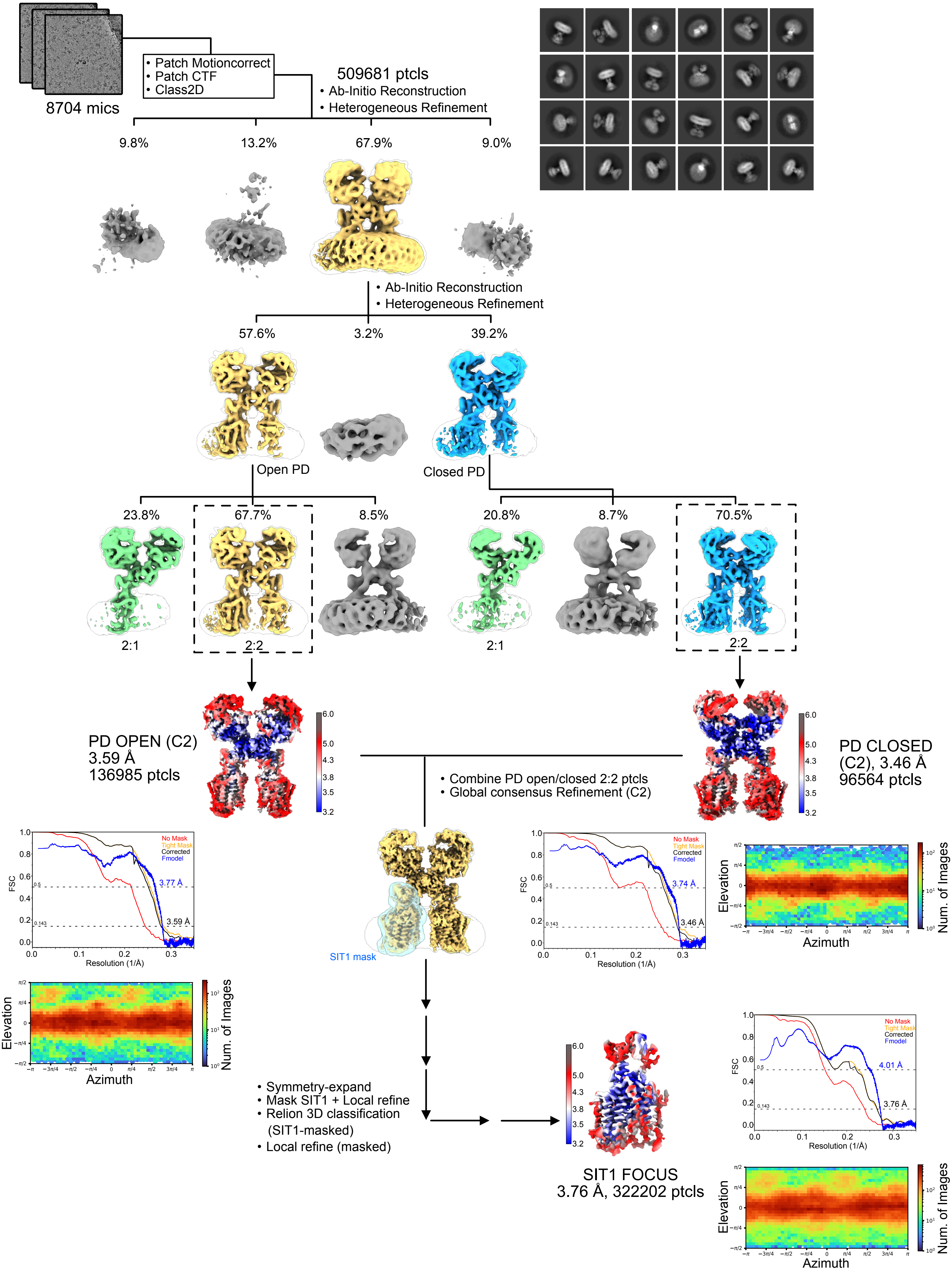

Supplementary Figure 5. ACE2-SIT1 apo structure determination. Flow chart for determining the structure of the ACE2-SIT1 complex in the presence of glycine.

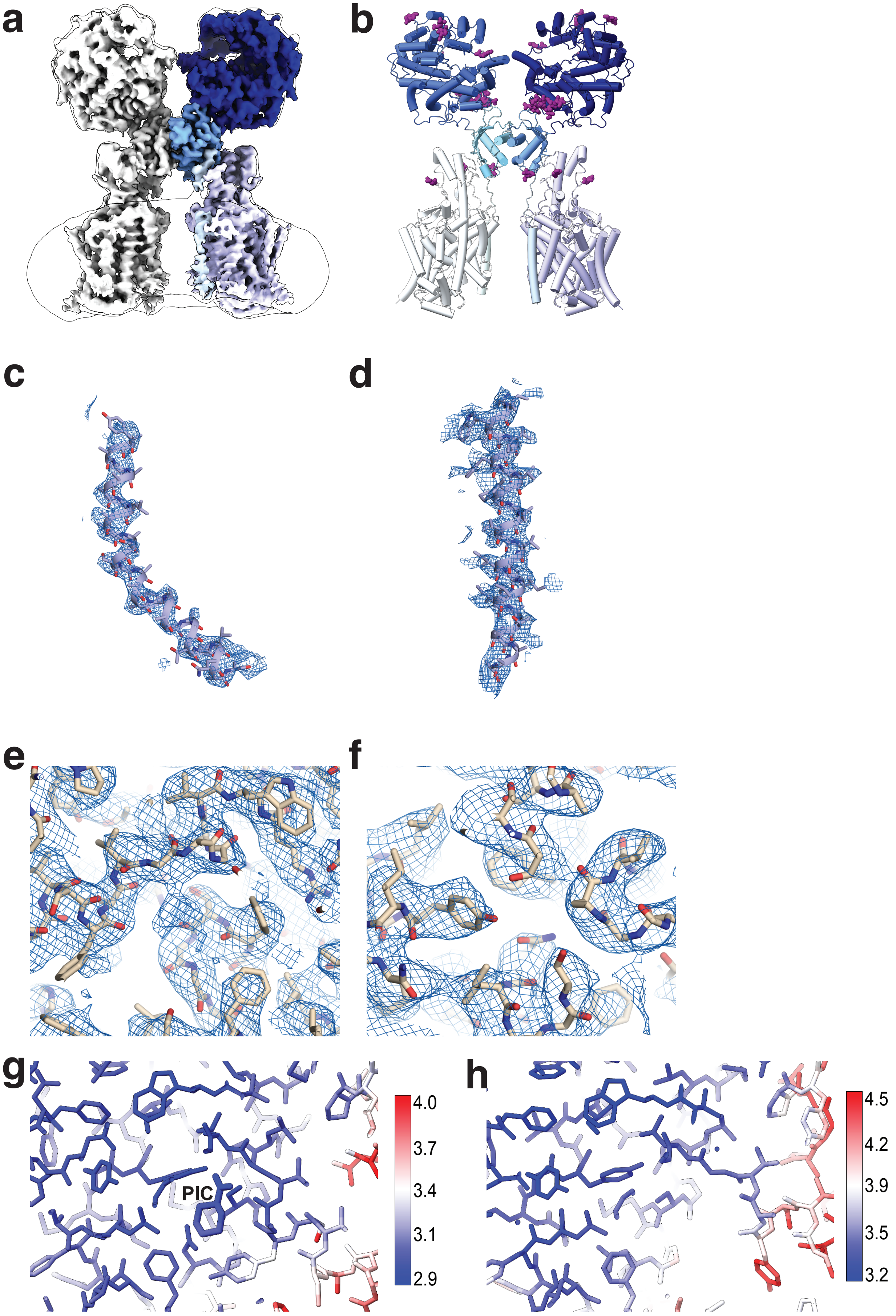

Supplementary Figure 6. SIT1-apo structure and binding sites. (**a**) Coulombic potential map and (**b**) experimental model for ACE2-SIT1 determined in the presence of glycine. Representative densities for (**c**) SIT1's TM12 and (**d**) ACE2's TMD. (**e**) Amino acid and (**f**) chloride binding sites of SIT1, determined in the presence of glycine. Coulombic potential map shown as blue mesh, protein shown as sticks. SIT1 binding site structure determined (**g**) bound to pipecolate or (**h**) in the presence of glycine, colored by the local resolution of the cryo-EM map.

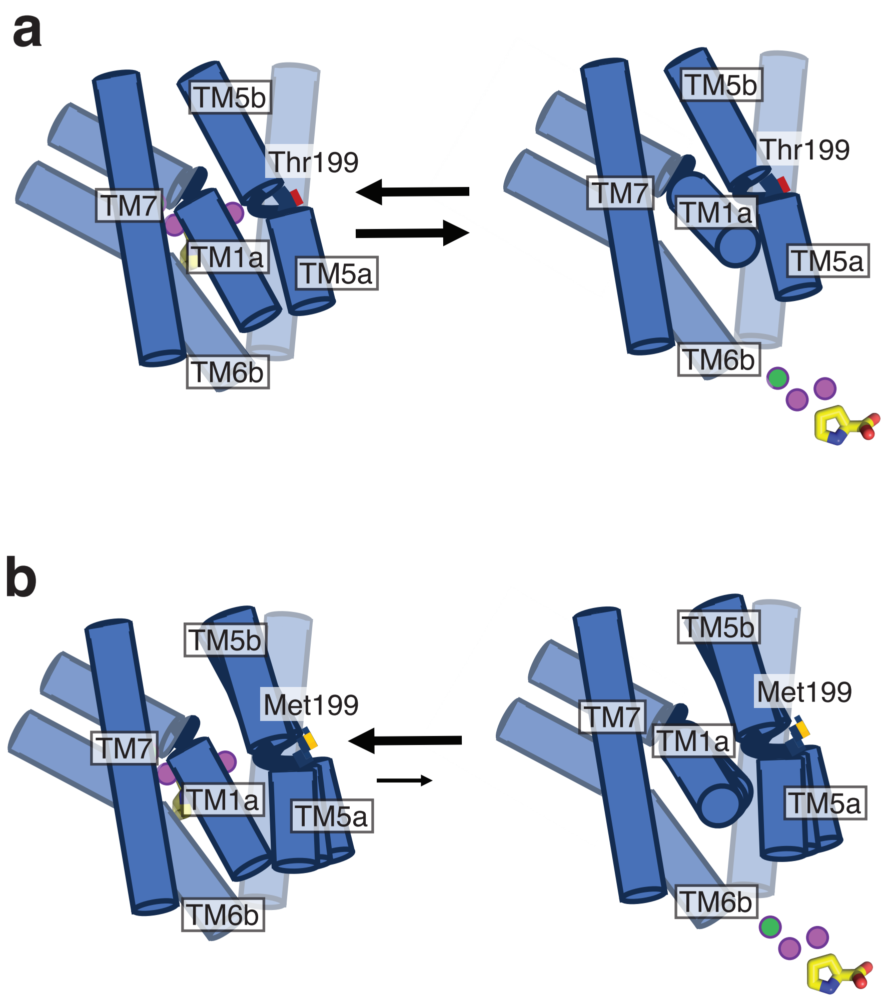

Supplementary Figure 7. Mechanism for SIT1 substrate release. (**a**) During opening of the cytoplasmic gate, TM1a rotates away from TM6a and TM7 to make contact with TM5b, thereby allowing substrate, sodium and chloride release. (**b**) The mutation T199M causes mispacking of TM5, and consequently the alters the position and energetics of that helix's interactions with TM1a in the open state. This destablizes the inward-facing open state of SIT1. Substrate proline, Thr199, and Met199 are shown as sticks, and ions are shown as spheres.
