# Supplementary material for: Structure and function of the SIT1 proline transporter in complex with the COVID-19 receptor ACE2
Source: Nat Commun. 2024 Jun 29;15:5503. doi: 10.1038/s41467-024-48921-x (PMC11217458; doi:10.1038/s41467-024-48921-x)
Supplement: Supplementary file 3 — Reporting Summary [file 41467_2024_48921_MOESM3_ESM.pdf]

nature portfolio

Corresponding author(s): Robert J. Vandenberg, Timothy R. Dafforn, Elisabeth P. Carpenter, David B. Sauer

Last updated by author(s): Apr 21, 2024

Reporting Summary

Nature Portfolio wishes to improve the reproducibility of the work that we publish. This form provides structure for consistency and transparency in reporting. For further information on Nature Portfolio policies, see our Editorial Policies and the Editorial Policy Checklist.

Statistics

For all statistical analyses, confirm that the following items are present in the figure legend, table legend, main text, or Methods section.

n/a	Confirmed	
☐	☒	The exact sample size (*n*) for each experimental group/condition, given as a discrete number and unit of measurement
☐	☒	A statement on whether measurements were taken from distinct samples or whether the same sample was measured repeatedly
☐	☒	The statistical test(s) used AND whether they are one- or two-sided<br>*Only common tests should be described solely by name; describe more complex techniques in the Methods section.*
☒	☐	A description of all covariates tested
☒	☐	A description of any assumptions or corrections, such as tests of normality and adjustment for multiple comparisons
☐	☒	A full description of the statistical parameters including central tendency (e.g. means) or other basic estimates (e.g. regression coefficient) AND variation (e.g. standard deviation) or associated estimates of uncertainty (e.g. confidence intervals)
☐	☒	For null hypothesis testing, the test statistic (e.g. *F*, *t*, *r*) with confidence intervals, effect sizes, degrees of freedom and *P* value noted<br>*Give P values as exact values whenever suitable.*
☒	☐	For Bayesian analysis, information on the choice of priors and Markov chain Monte Carlo settings
☒	☐	For hierarchical and complex designs, identification of the appropriate level for tests and full reporting of outcomes
☒	☐	Estimates of effect sizes (e.g. Cohen's *d*, Pearson's *r*), indicating how they were calculated

Our web collection on statistics for biologists contains articles on many of the points above.

Software and code

Policy information about availability of computer code

Data collection	Cryo-EM data were collected with EPU (version 2.13; Thermo Fisher Scientific).
Data analysis	Cryo-EM data were processed and analyzed with cryoSPARC (version 3.3.1; Structura Biotechnology) and Relion (version 3). Structural models were built using Coot (v0.8.9.5) with model refinement performed by PHENIX (v1.20).<br><br>Molecular visualisation, analysis and figure generation were performed using PyMOL 2.2.0 (Schrodiner), ChimeraX 1.16 (UCSF Resource for Biocomputing, Visualization, and Informatics), and Prism 10.1.1 (GraphPad)

For manuscripts utilizing custom algorithms or software that are central to the research but not yet described in published literature, software must be made available to editors and reviewers. We strongly encourage code deposition in a community repository (e.g. GitHub). See the Nature Portfolio guidelines for submitting code & software for further information.

Data

Policy information about availability of data

All manuscripts must include a data availability statement. This statement should provide the following information, where applicable:

- Accession codes, unique identifiers, or web links for publicly available datasets
- A description of any restrictions on data availability
- For clinical datasets or third party data, please ensure that the statement adheres to our policy

The cryo-EM maps and models generated in this study have been deposited in the EMDB database and the Protein Data Bank, respectively, under accession codes, EMD-17381 and PDB-8P30 for ACE2-SIT1 with pipecolate bound and open peptidase domain, EMD-17382 and PDB-8P31 for ACE2-SIT1 with pipecolate bound and closed peptidase domain, EMD-17380 and PDB-8P2Z for SIT1 with pipecolate bound focused refinement, EMD-17378 and PDB-8P2X for ACE2-SIT1 with open peptidase domain determined in the presence of glycine, EMD-17379 and PDB-8P2Y for ACE2-SIT1 with closed peptidase domain determined in the presence of glycine, EMD-17377 and PDB-8P2W for SIT1 focused refinement determined in the presence of glycine.

Research involving human participants, their data, or biological material

Policy information about studies with human participants or human data. See also policy information about sex, gender (identity/presentation), and sexual orientation and race, ethnicity and racism.

Reporting on sex and gender	*NA*
Reporting on race, ethnicity, or other socially relevant groupings	*NA*
Population characteristics	*NA*
Recruitment	*NA*
Ethics oversight	*NA*

Note that full information on the approval of the study protocol must also be provided in the manuscript.

Field-specific reporting

Please select the one below that is the best fit for your research. If you are not sure, read the appropriate sections before making your selection.

☒ Life sciences ☐ Behavioural & social sciences ☐ Ecological, evolutionary & environmental sciences

For a reference copy of the document with all sections, see nature.com/documents/nr-reporting-summary-flat.pdf

Life sciences study design

All studies must disclose on these points even when the disclosure is negative.

Sample size	No statistical method was used to predetermine sample size. The transport and binding study samples sizes of N=S and N=3, respectively, were chosen to ensure reproducibility based on prior experimental experience.
Data exclusions	None
Replication	Cryo-EM data collection was only carried out on a single sample in each case. All in vivo experiments used 5 biological replicates. In vitro binding assays were carried out with 3 biological replicates. All replicates were successful.

Randomization	For cryo-EM studies, particles were randomly assigned to half-maps for resolution determination. No randomization was not used for transport or inhibition assays.
Blinding	Blinding was not used. It is not relevant as outcomes of the experiments are not affected by knowledge of the sample.

Reporting for specific materials, systems and methods

We require information from authors about some types of materials, experimental systems and methods used in many studies. Here, indicate whether each material, system or method listed is relevant to your study. If you are not sure if a list item applies to your research, read the appropriate section before selecting a response.

Materials & experimental systems

n/a	Involved in the study
☒	☐ Antibodies
☐	☒ Eukaryotic cell lines
☒	☐ Palaeontology and archaeology
☐	☒ Animals and other organisms
☒	☐ Clinical data
☒	☐ Dual use research of concern
☒	☐ Plants

Methods

n/a	Involved in the study
☒	☐ ChIP-seq
☒	☐ Flow cytometry
☒	☐ MRI-based neuroimaging

Eukaryotic cell lines

Policy information about cell lines and Sex and Gender in Research

Cell line source(s)	Spodoptera frugiperda (Sf9) cells (Thermo-Fisher Scientific, Cat. No. 11496015). Expi293F GnTI- cells (Cat# A39240; Thermo Fisher Scientific).
Authentication	Cell lines used (SF9/Expi293F/HEK293-JI) are standard laboratory model overexpression strains purchased from Thermo Fisher. These cell lines undergo quality control before dispatch. Cells were passaged a limited number of times before a new batch from the manufacturer was employed. Cells were monitored by regular visual inspection.
Mycoplasma contamination	Not performed.
Commonly misidentified lines (See ICLAC register)	No commonly misidentified cell lines were used in this study.

Animals and other research organisms

Policy information about studies involving animals; ARRIVE guidelines recommended for reporting animal research, and Sex and Gender in Research

Laboratory animals	Xenopus laevis. Animals themselves were not used. As described under Methods, oocytes were harvested from adult female frogs that were between 1-5 years.
Wild animals	No wild animals were used in this study.
Reporting on sex	Xenopus laevis for oocyte collection were female.
Field-collected samples	No field collected samples were used in this study.
Ethics oversight	Xenopus laevis oocytes were supplied by the Victor Chang Cardiac Research Institute and approved by the Garvan Institute/St Vincents Hospital Animal Ethics Committee (Animal Research Authority 23_11).

Note that full information on the approval of the study protocol must also be provided in the manuscript.

Plants

Seed stocks	*Report on the source of all seed stocks or other plant material used. If applicable, state the seed stock centre and catalogue number. If plant specimens were collected from the field, describe the collection location, date and sampling procedures.*
Novel plant genotypes	*Describe the methods by which all novel plant genotypes were produced. This includes those generated by transgenic approaches, gene editing, chemical/radiation-based mutagenesis and hybridization. For transgenic lines, describe the transformation method, the number of independent lines analyzed and the generation upon which experiments were performed. For gene-edited lines, describe the editor used, the endogenous sequence targeted for editing, the targeting guide RNA sequence (if applicable) and how the editor was applied.*
Authentication	*Describe any authentication procedures for each seed stock used or novel genotype generated. Describe any experiments used to assess the effect of a mutation and, where applicable, how potential secondary effects (e.g. second site T-DNA insertions, mosiacism, off-target gene editing) were examined.*
