# Supplementary figures and images for: Structure and function of the SIT1 proline transporter in complex with the COVID-19 receptor ACE2
Source: Nat Commun. 2024 Jun 29;15:5503. doi: 10.1038/s41467-024-48921-x (PMC11217458; doi:10.1038/s41467-024-48921-x)

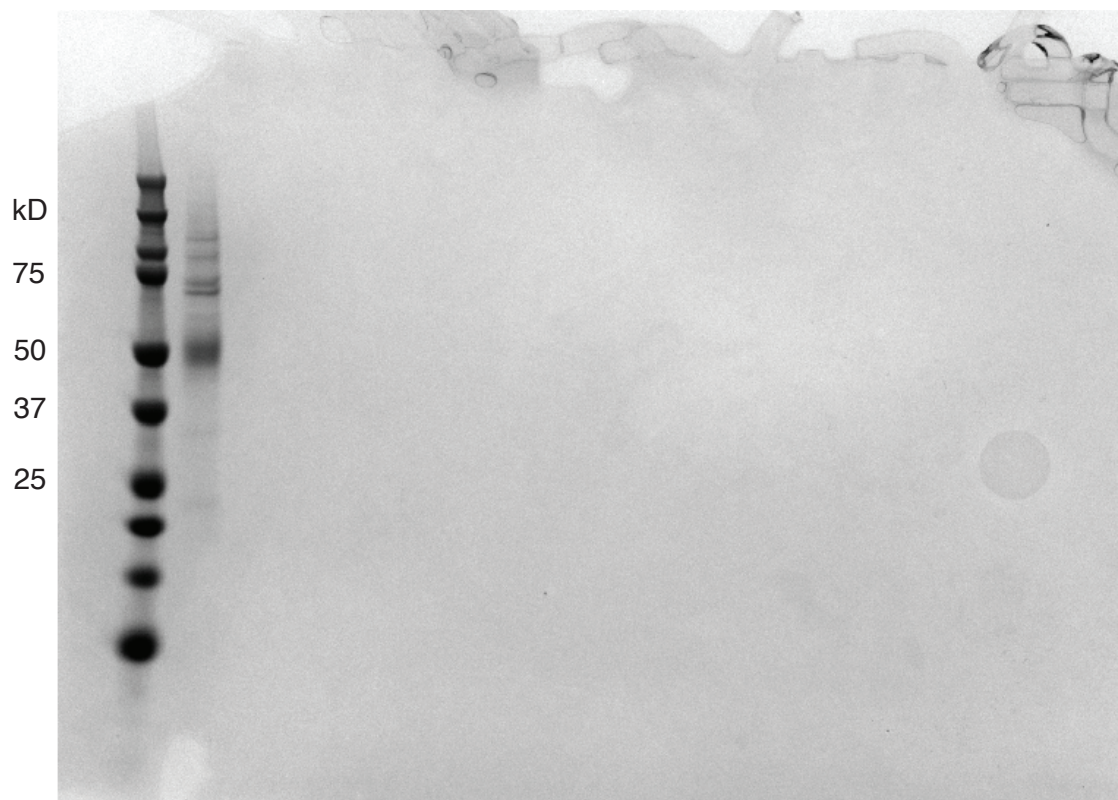

**Source data for Supplementary Figure 1c**

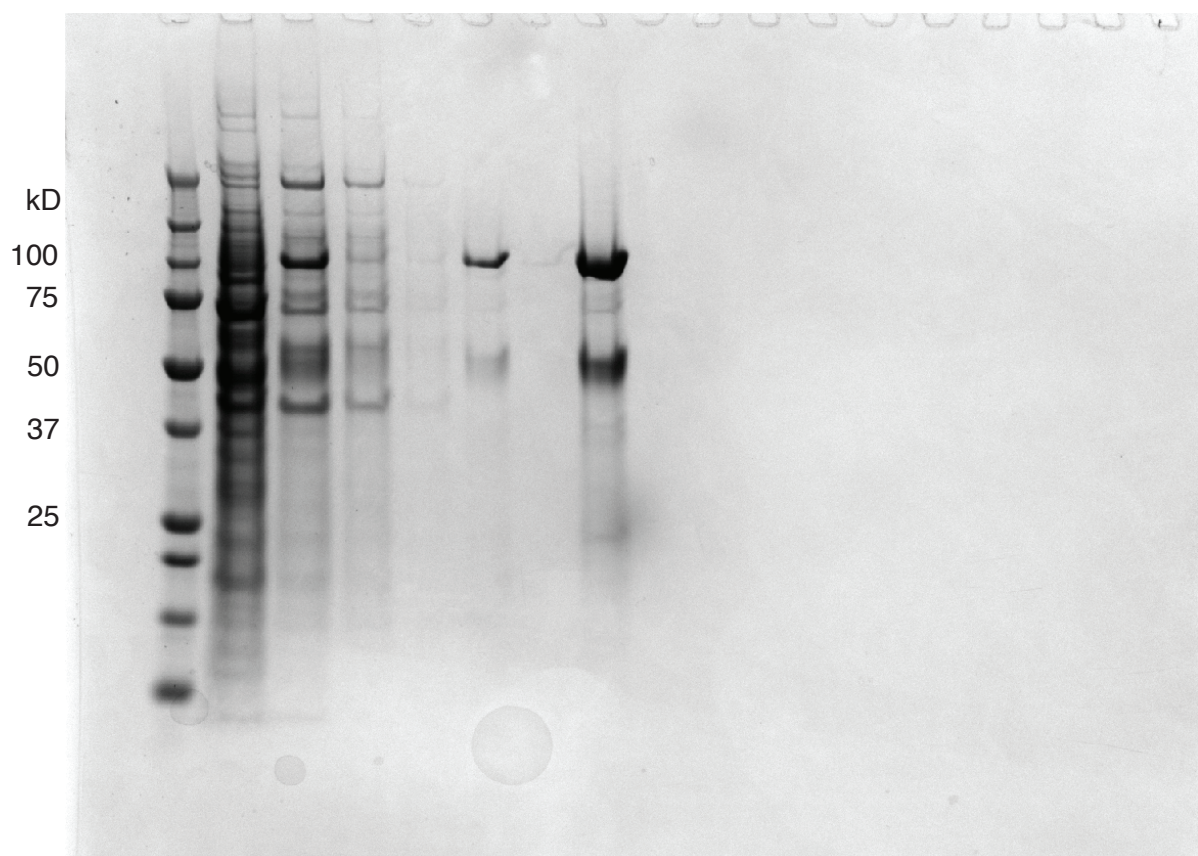

**Source data for Supplementary Figure 1f**

Supplement: Supplementary file 4 — Source Data [file 41467_2024_48921_MOESM4_ESM.zip › Source data/uncropped_gels.pdf]
